# Anti-inflammatory, expectorant, and antitussive properties of *Kyeongok-go* in ICR mice

**DOI:** 10.1080/13880209.2021.1892155

**Published:** 2021-03-26

**Authors:** Jin-Ryul Hu, Chul-Jong Jung, Seong-Min Ku, Dae-Hwa Jung, Khawaja Muhammad Imran Bashir, Sae-Kwang Ku, Jae-Suk Choi

**Affiliations:** a Department of Histology and Anatomy, College of Korean Medicine, Daegu Haany University, Gyeongsan-si, Republic of Korea; b Okchundang Inc, Ulsan, Republic of Korea; c Department of Pharmaceutical Engineering, Daegu Haany University, Gyeongsan, Republic of Korea; d German Engineering Research Center for Life Science Technologies in Medicine and Environment, Busan, Republic of Korea; e The Medical Research Center for Herbal Convergence on Liver Disease, Daegu Haany University, Gyeongsan-si, Republic of Korea; f Department of in Food Biotechnology, College of Medical and Life Sciences, Silla University, Busan, Republic of Korea

**Keywords:** Traditional mixed herb, respiratory disease, rodent experiment

## Abstract

**Context:**

*Kyeongok-go* (KOG) is a traditional mixed herb preparation consisting of *Panax ginseng* CA Meyer (Araliaceae), *Poria cocos* Wolf (Polyporaceae), *Rehmannia glutinosa* (Gaertner) Liboschitz ex Steudel (Orobanchaceae), and honey. Various pharmacological effects of KOG are reported, but the efficacy on respiratory diseases has not been evaluated.

**Objective:**

The anti-inflammatory, expectorant, and antitussive properties of KOG were examined using animal models of respiratory diseases.

**Materials and methods:**

KOG (100, 200, and 400 mg/kg) was orally administered to ICR mice (*n* = 8) once a day for 11 days. Anti-inflammatory effects of vehicle, xylene, KOG and DEXA (1 mg/kg) were determined by monitoring edoema and redness of treated ears, and measuring the relative and absolute weight of each ear. Expectorant properties of vehicle, KOG and AM (250 mg/kg) were evaluated by observing body surface redness, and the amount of mucous secreted by the trachea. The antitussive potential of vehicle, NH_4_OH, KOG and TB (50 mg/kg) was evaluated by monitoring changes in the number of coughs (for 6 min).

**Results:**

KOG (400 mg/kg) treated mice showed 31.29% and 30.72% (*p* < 0.01) decreases in the relative and absolute weights of each ear relative to xylene control mice, 39.06% increases (*p* < 0.01) in TLF OD values relative to intact vehicle control mice, and 59.53% decrease (*p* < 0.01) in coughing compared to NH_4_OH control mice. Dose-dependent changes were observed in all experimental models.

**Conclusions:**

KOG may be a potential therapeutic agent for the treatment of various respiratory diseases, particularly those caused by environmental toxins.

## Introduction

Cough is defined as a forced expulsive manoeuvre, usually against a closed glottis and is associated with a characteristic sound (Morice et al. 2007). Cough can be the result of several respiratory tract disorders that may require drug treatment for relief. A patient's quality of life could significantly be affected by chronic cough (Birring et al. 2003). The intrapulmonary rapidly adapting receptor, RAR, a cough receptor causes or enhances the sensitivity to stimuli that result in a cough (Pavord 2004). RAR activation initiates bronchospasm and mucus secretion via parasympathetic reflexes (Dapaah et al. 2016). Cough can be described as non-productive (dry) or productive (chesty) (Dapaah et al. 2016). Antitussives are effective in managing a non-productive cough but are not as effective at alleviating a productive cough, except when the antitussive has expectorant properties (Dicpinigaitis and Gayle 2003; Sanak 2016; Woloski et al. 2016). Therefore, it is thought that drugs with simultaneous anti-inflammatory, expectorant, and antitussive activities are protective against various respiratory disorders (Li et al. 2012; Wang et al. 2012; Yu et al. 2015). Recently, several pharmacological agents were shown to have both antitussive and expectorant effects making them useful for the treatment of both a chesty and a dry cough (Dicpinigaitis and Gayle 2003). The importance of this dual effect is affirmed using both antitussives and expectorants in pharmaceutical formulations (Dapaah et al. 2016).

Currently, coughing can be alleviated with the use of drugs such as antitussives and expectorants such as codeine, theobromine (TB), and ambroxol (AM); however, treatment should encompass the underlying condition that induces the cough. The problem is more than just efficient therapy for coughing, as this will inevitably result in side effects. TB, a bitter alkaloid of the cacao plant, has shown higher antitussive effects by suppressing vagus nerve activity (Usmani et al. 2005; Halfdanarson and Jatoi 2007). However, consumption of TB in large quantities has been associated with TB poisoning, especially in the elderly (Sutton 1981; Derlet et al. 1992). AM, a secretolytic agent, is used in the treatment of respiratory diseases caused by excessive mucus or viscid (Beeh et al. 2008; Malerba and Ragnoli 2008). However, patients with gastric ulceration are cautioned against its use, and usage of AM during the first trimester of pregnancy is not recommended (Molina et al. 2004). Adrenocorticosteroids, including dexamethasone (DEXA) have been reported with anti-allergic and anti-inflammatory properties (Kim et al. 2010; Ku et al. 2012); however, the use of adrenocorticosteroids causes serious side effects such as foetal immunodeficiency (Tasaka 1986). Therefore, there is an increasing demand for anti-inflammatory, expectorant, and antitussive therapy for the treatment of various respiratory disorders (Li et al. 2012; Wang et al. 2012; Yu et al. 2015).

*Kyeongok-go* (KOG) is a traditional mixed herb preparation consisting of fresh root parts of *Panax ginseng* C. A. Meyer (Araliaceae), grounded powders of *Poria cocos* Wolf (Polyporaceae), fresh root parts of *Rehmannia glutinosa* (Gaertner) Liboschitz ex Steudel (Orobanchaceae) and honey; used as a vitalizing tonic in Korea (Kim and Song 2014; Na et al. 2016). Various pharmacological effects of KOG, including hypolipidemic (Kim and Song 2014), antioxidant (Na et al. 2016), immunomodulatory (Na et al. 2016), anti-inflammatory (Lee et al. 2013), and anti-osteoporotic effects (Kim, Lee et al. 2011), as well as hair growth promotion (Do et al. 2011), antifatigue, and aerobic capacity enhancement (Kim, Park et al. 2011), growth promotion (Cha 2009), anti-aging (Kwak 2003), and antibacterial (Jeon 2000) effects have been documented.

In this study, the anti-inflammatory, expectorant, and antitussive properties of KOG were analyzed using the following animal models: (a) NH_4_OH-induced cough mouse model was utilized as the antitussive assay (Zhang et al. 2009; Wang et al. 2012), (b) tracheal phenol red secretion method was used to determine the expectorant effect of KOR (Engler and Szelenyi 1984; Zhang et al. 2009; Wang et al. 2012), and (c) the xylene-induced acute inflammatory mouse ear model was used to assess the anti-inflammatory effects of KOG (Cho et al. 2008; Lee and Ku 2008). KOG (100, 200, and 400 mg/kg), was orally administered to mice once a day for 11 days, and the results were compared with control treatments.

## Materials and methods

### Test substances

KOG, a black coloured viscous material, was purchased from Okchundang (Ulsan, Republic of Korea). Briefly, appropriate amounts of each herb powder-Ginseng *Radix alba* (6000 g), *Poria cocos* (Schw.) Wol (12,000 g), and *Rehmanniae radix* Crudus (47,000 g) and honey (39,000 g) were mixed and heated in a water bath at 60 °C for 72 h and cooled at room temperature, twice repeatedly (Table 1). Some specimens of KOG were deposited in the herbarium of the Medical Research Centre for Globalisation of Herbal Formulation, Daegu Haany University (Gyeongsan, Rep. of Korea) [Reference No: KOG2016Ku01]. In addition, DEXA, a water-soluble white granule, and AM and TB in the form of a white powder, purchased from Sigma-Aldrich (St. Louis, MO) were used as reference drugs in anti-inflammatory, expectorant, and antitussive assays, respectively. KOG, TB, AM, and DEXA were all stored at 4 °C protected from light to prevent degradation until use.

**Table 1.** Composition of KOG used in this study.

Herbs	Scientific names	Production distinct	Amounts (g)
Ginseng Radix Alba	*Panax ginseng* C. A. Meyer	Korea	6000
Pulvis Hoelen	*Poria cocos* Wolf	China	12,000
Honey		Korea	39,000
Rehmanniae Radix Crudus	*Rehmannia glutinosa* (Gaertner) Liboschitz ex Steudel	Korea	47,000
Total	4 types		104,000

Individual herbs were prepared by Okchundang (Ulsan, Korea).

KOG: *Kyeongok-go*, Traditional mixed herbal formulation.

### Analysis of specific ingredients of KOG

#### Instruments and reagents

ACQUITY^TM^ Ultra Performance Liquid Chromatography (UPLC) system equipped with ACQUITY^TM^ UPLC Photodiode Array Detector (PDA; Waters Corp, Milford, MA), and ACQUITY^TM^ BEH C_18_ column (1.7 μm, 2.1 × 100) were used for UPLC analysis. In addition, Empower Chromatography Data software (Waters Corp, Milford, MA) was used to analyze the results. The sample was extracted with ultrasonicator (Branson Ultrasonics, Danbury, CT). The reagents, methanol (Junsei Chemical Co. Ltd., Tokyo, Japan), acetonitrile (BAKER, Centre Valley, PA), and water (tertiary distilled water) were all HPLC grade. The standard preparations were purchased from Extrasynthese (Genay Cedex, France) or Sigma-Aldrich (St. Louis, MO).

#### Preparation of the standard solution

Rehmanniae Radix Crudus containing acteoside, catalposide, and 5-hydroxymethyl-2-furfural (5H3F), and Ginseng Radix Alba containing (Ginsenoside Rg3 [Rg3]) were measured and dissolved in methanol to prepare stock concentrations of 1 μg/mL per solution. Varying amounts of each solution were diluted in methanol to obtain 1, 5, 10 ng/mL of the standard solution. The coefficient of determination was R^2^ > 0.999, for all solutions tested.

#### Test liquid preparation for quantitative analysis

The test liquid for quantitative analysis was prepared by dissolving equal amounts of each sample (1 g) in 10 mL of 30% methanol. Extracts were removed by microwave for 1 h. The extract was filtered by a < 0.2 μm membrane filter and used as the test liquid.

#### Quantitation of the ingredients

**Figure 1.**
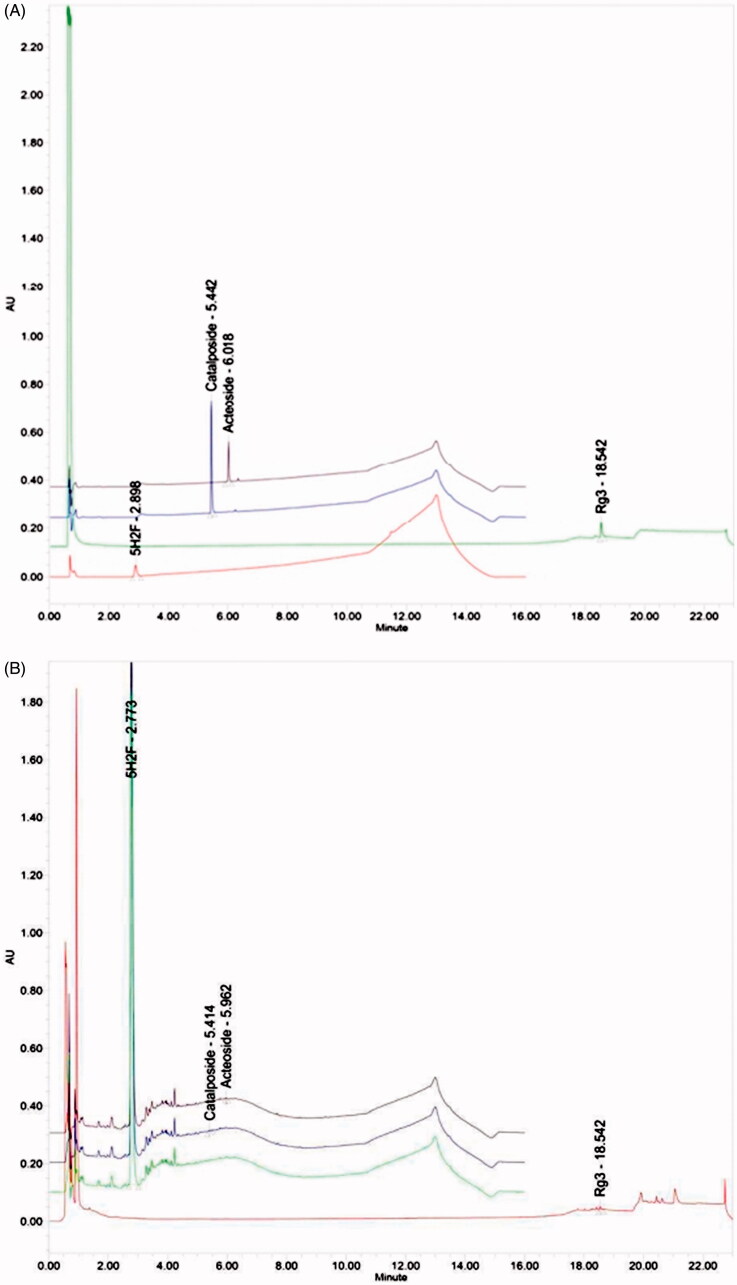
UPLC analysis of chemical standards (A) and specific ingredients of KOG (B). KOG: *Kyeongok-go*, Traditional mixed herbal formulation; UPLC: Ultra performance liquid chromatography; 5H2F: 5-hydroxymethyl-2-furfural; Rg3: Ginsenoside Rg3.

The amount of acteoside, catalposide, 5H3F, and Rg3 in KOG were all quantified using UPLC equipped with a PDA, BEH C_18_ column, and the Empower software. The column was kept at the room temperature during analysis. Acteoside, catalposide, and 5H3F were analyzed at 280 nm, whereas Rg3 was analyzed at 203 nm. A mobile phase consisting of a mixture of acetonitrile, and water containing 0.1% formic acids, was produced by all the samples. The analysis conditions were as follows: 2 μL of each sample was injected at a flow rate of 0.4 mL/min, and the results were qualitatively analyzed by determining the retention time and quantified using the peak area method (Figure 1).

#### Animals husbandry

One hundred thirty-six 6-week-old male specific pathogen free/virus antibody free CrljOri:CD1, ICR-mice weighing 29–32 g were obtained from Orient Bio (Seungnam, Rep. of Korea) and acclimatized for 7 d. The acclimatized mice were divided into six groups of eight mice each for antitussive and anti-inflammatory assay and five groups of eight mice each for expectorant assay, based on their body weights [intact control: 34.89 ± 1.39 g, NH_4_OH treated mice: 34.11 ± 1.45 g]. Groups containing four mice each were placed into polycarbonate cages in a humidity (50–55%) and temperature (20–25 °C) controlled room. The light was provided for 12 h. Standard rodent chow (Purinafeed, Seungnam, Rep. of Korea) and water were freely accessible.

All laboratory animals were treated according to the national regulations on the usage and welfare of laboratory animals and were approved by the Institutional Animal Care and Use Committee, Daegu Haany University Gyeongsan, Gyeongbuk, Rep. of Korea [Reference No: DHU2016-034].

### Anti-inflammatory assay

**Experimental groups** (Six groups containing eight mice each, eight mice in each group were later sacrificed):

Intact vehicle control: vehicle (distilled water) treated intact control mice.

Xylene control: vehicle treated and xylene topically applied control mice.

DEXA: DEXA (1 mg/kg; Hu et al. 2019) treated and xylene topically applied mice.

KOG100: KOG (100 mg/kg) treated and xylene topically applied mice.

KOG200: KOG (200 mg/kg) treated and xylene topically applied mice.

KOG400: KOG (400 mg/kg) treated and xylene topically applied mice.

#### Measurement of body weight

Changes in body weight were measured once a day starting one day before treatment to the end of treatment on day 1, using an automatic laboratory animal balance (Precisa Instrument, Dietikon, Switzerland). Animals were fasted overnight (up to 18 h, however, water was freely provided) before the initial administration of each test substance. Mice were killed to reduce individual differences in feeding, and to reduce the individual difference in body weight at the start of the experiment. The weight gained during the 11 d treatment period of KOG or TB were calculated as depicted in Equation 1.

(1) Weight gained during the 11 d treatment period of test substances = [Body weight at sacrifice (g)]− [Body weight at initial treatment (g)]

#### Administration of test substance

KOG was orally administered once a day for 11 d before the topical application of xylene, following the protocol described above in the antitussive assay. In addition, DEXA (1.5 mg/mL, 0.1 mg/mL based on DEXA itself) was orally administered in a volume of 10 mL/kg (equivalent to 1 mg/kg based on DEXA itself), once a day for 11 d of xylene treatment. For xylene control and intact vehicle control mice, distilled water was orally administered (10 mL/kg), instead of DEXA or KOG, to mimic the restraint stresses.

#### Induction of acute inflammation

Acute inflammation was induced by a single topical application of 0.03 mL of xylene (Duksan Pure Chemical Co. Ltd., Ansan, Rep. of Korea) to the anterior surface of the right ear 1 h after administration of last test substance (day 11), as described previously (Cho et al. 2008; Lee and Ku 2008) with some modifications. An equal volume of saline was topically applied to the ears of intact vehicle mouse, instead of xylene.

#### Measurement of ear weight

Circular sections of each ear were isolated using a 7 mm diameter-cork borer after 2 h of topical application of xylene and absolute wet-weights were measured. The relative weight (% of body weight) of each ear was calculated to normalize for the differences in body weight among individual mice. Equation 2 was used to calculate relative ear weight.

(2) Relative ear weight (% of body weights)= [Absolute ear wet weight (g)/body weight at sacrifice (g)] × 100

#### Histopathology

After the weight measurements, ear samples were fixed in 10% neutral-buffered formalin (NBF), and crossly trimmed, embedded in paraffin, sectioned (3–4 μm), and stained with haematoxylin-eosin (H&E) for general histopathology, or toluidine blue for mast cell analysis. Histopathological analyses were performed under a light microscope. To point out the changes, the following were measured: (a) mean total dermis and epidermis thickness of the ear anterior surface, (b) Infiltrating inflammatory cells and mast cells numbers on the dermis of ear (cells/mm^2^), and (c) the collagen occupied regions percentage on the dermis (%/mm^2^), and were analyzed using a computer-assisted image analysis program (Cho et al. 2008; Lee and Ku 2008; Lee et al. 2014; Kim, Kang et al. 2015, Kim, Park et al. 2015). Furthermore, the histopathologist was blinded and at least five repeated measurements on same histological specimens were performed to draw the mean histomorphometrical data.

### Expectorant assay

**Experimental groups** (Five groups containing eight mice each):

Control: vehicle (distilled water) treated intact control mice.

AM: AM (250 mg/kg; Hu et al. 2019) treated mice.

KOG100: KOG (100 mg/kg) treated mice.

KOG200: KOG (200 mg/kg) treated mice.

KOG400: KOG (400 mg/kg) treated mice.

#### Measurement of body weight

Changes in body weight were calculated as described above in the anti-inflammatory assay.

#### Administration of test substance

KOG was orally administered once a day for 11 d before phenol red treatment, following the protocol described above in the antitussive assay. In addition, AM (25 mg/mL) was orally administered at a dose of 10 mL/kg (equivalent to 250 mg/kg), once a day for 11 d before phenol red treatment. For intact vehicle control mice, 10 mL/kg distilled water was orally administered, instead of KOG or AM, to mimic restraint stress.

#### Measurement of mucous secretions

Mucosal secretions were calculated using the tracheal phenol red secretion assay. A single intraperitoneal injection of 5% phenol red (Junsei Chemical Co. Ltd., Tokyo, Japan) in saline (*w*/*v*; 10 mL/kg) was administered 30 min after the last treatment with a test substance (day 11). Thirty min post phenol red injection, all mice were killed by cervical dislocation without damaging the trachea, gross images were acquired to monitor redness on body surface. After isolation from adjacent organs, the trachea was removed from the main stem bronchi and the thyroid cartilage and ultrasonicated for 15 min (Branson Ultrasonics, Danbury, CT). After sonication, 1 mL of 5% NaHCO_3_ was add to the normal saline, and the optical density of the prepared tracheal lavage fluid (TLF) was measured at 546 nm (Tecan, Männedorf, Switzerland; Engler and Szelenyi 1984; Zhang et al. 2009; Wang et al. 2012).

#### Histopathology

In parallel, lungs (left lateral lobes) were isolated during trachea excision, fixed with 10% NBF, and crossly trimmed. The tissues were embedded in paraffin, sectioned (3–4 μm), and stained with H&E for general histopathology, or periodic acid Schiff (PAS) to detect mucous producing cells. Light microscope was used to observe each histopathological finding. To analyze detailed changes the following were measured: (a) secondary bronchus mucosal mean thickness, and (b) the PAS positive mucous producing cells number on the secondary bronchus (cells/mm^2^), and were examined using a computer-assisted image analysis program (Tasaka 1986; Choi et al. 2005; Tam et al. 2014; Honda 2016). The histopathologist was blinded to group distributions and at least five repeated examinations on same histological specimens were conducted to calculate each mean histomorphometrical data.

### Antitussive assay

***Experimental groups*** (Six groups containing eight mice each).

Intact vehicle control: vehicle (distilled water) treated intact control mice.

NH_4_OH control: vehicle treated and NH_4_OH-exposed control mice.

TB: TB (50 mg/kg; Hu et al., 2019) treated and NH_4_OH-exposed mice.

KOG100: KOG (100 mg/kg) treated and NH_4_OH-exposed mice.

KOG200: KOG (200 mg/kg) treated and NH_4_OH-exposed mice.

KOG400: KOG (400 mg/kg) treated and NH_4_OH-exposed mice.

#### Measurement of body weight

Changes in body weight were calculated as described above in the anti-inflammatory assay.

#### Administration of test substance

KOG was resuspended in distilled water at concentrations of 10, 20, and 40 mg/mL, and orally administered in a volume of 10 mL/kg (equivalent to 100, 200, and 400 mg/kg), once a day for 11 d before exposure to NH_4_OH. Similarly, TB was dissolved in distilled water at a concentration of 5 mg/mL, and orally administered at a dose of 10 mL/kg (equivalent to 50 mg/kg), once a day for 11 d, before NH_4_OH exposure. Control mice, intact vehicle, and NH_4_OH control, were orally administered 10 mL/kg of distilled water (instead of KOG or TB), in order to mimic the stress associated with restraining the animals.

#### Induction of cough and monitoring

Cough was induced by single inhalation of 25% NH_4_OH (Sigma-Aldrich, St. Louis, MO); 0.3 mL of 25% NH_4_OH in a 1000 mL glass Erlenmeyer flask was individually administered for 45 sec, 1 h after the last treatment of each test substance on day 11. After NH_4_OH exposure, the number of coughs were recorded and measured during a 6 min observation period; video observations were conducted as previously described (Zhang et al. 2009; Wang et al. 2012) with slight modifications. Individual intact vehicle control mice were subjected to 0.3 mL of saline in a 1,000 mL glass Erlenmeyer flask for 45 sec, instead of NH_4_OH. The cough in mice was defined by opening of the mouth and the accompanying sound of coughing, contraction of thoracic and abdomen muscles, and abdominal jerking (Zhang et al. 2009; Wang et al. 2012).

#### Histopathology

After acquiring video and images, individual lungs (left lateral lobes) and trachea (3 mm from thyroid cartilages) were sampled and fixed in 10% NBF, and crossly trimmed. The tissue was embedded in paraffin, sectioned (3–4 μm), and stained with haematoxylin and eosin (H&E) for general histopathology. Toluidine blue was used to detect mast cells, and light microscope (Nikon, Tokyo, Japan) was used for histopathological profile analyses. In order to point the changes, the following parameters were used; (a) the mean diameter of the tracheal lumen (μm), (b) thickness of the submucosa, tracheal wall, and epithelium (μm) (c) number of mast on the trachea and infiltrating inflammatory cells (cells/mm^2^), (d) mean alveolar surface area (ASA) (%/mm^2^), (e) mean thickness of alveolar septum (μm), and (f) the number of infiltrating inflammatory cells in the alveolar septum (cells/mm^2^). These parameters were analyzed using a computer-assisted image analysis program, *i*Solution FL ver 9.1 (IMT *i*-solution Inc., Quebec, Canada), as suggested by the previously reported studies (Lebargy et al. 1987; Choi et al. 2005; Ku et al. 2012). The histopathologist was blinded to the group distributions, and at least five repeated measurements on the same histological specimens were performed to calculate mean histomorphometrical data.

### Statistical analyses

The experimental data are expressed as mean ± standard deviation (SD) of six independent readings of eight mice. Different dose groups were compared using multiple comparison tests. Levene test was used to examine the variance homogeneity (Levene 1981). In case of no significant deviations, the data were analyzed by one-way ANOVA followed by the least-significant differences multi-comparison (LSD) test to define the significantly different groups. In case of a significant deviations, the Kruskal-Wallis H test (a non-parametric comparison test) was performed. In case of a significant difference observed by the Kruskal-Wallis H test, the significantly different groups were defined using the Mann–Whitney U (MW) test. SPSS Ver. 14 (IBM SPSS Inc., Armonk, NY) was used to conduct the statistical analyses (Ludbrook 1997) and the differences were considered significant at *p* < 0.05. Furthermore, the percentage change in between induced and intact vehicle controls was calculated to determine the severity of the formed lesions. These percent differences between induced controls and test substance treated mice were also calculated as a measure of efficacy using Equations 3 and 4 following the previously reported study (Kang et al. 2014).

(3) Percent change compared to intact vehicle control (%) =[(Data of induced control − Data of intact vehicle control mice)  /Data of intact vehicle control mice] × 100

(4) Percent change compared to induced control (%)=[(Data of test substance treated mice − Data of induced control mice)/Data of induced control mice] × 100

## Results

### Contents of specific ingredients of KOG

UPLC analysis of KOG used in this study showed 5-hydroxymethyl-2-furfural (5H2F), acteoside, catalposide, and ginsenoside Rg3 (Rg3) were detected at 628.26 ± 13.20, 0.330 ± 0.02, 0.409 ± 0.03 and 7.267 ± 0.46 mg/kg concentrations, respectively (Figure 1).

### Anti-inflammatory assay

#### Body weight changes

No significant differences in body weight were detected in between the intact vehicle control and xylene control mice. In addition, KOG 100, 200, and 400 mg/kg treated mice and xylene control mice had similar body weights during the treatment period. However, the weight of DEXA treated mice significantly (*p* < 0.05) decreased 2 and 3 d after initial administration relative to intact vehicle and xylene control mice. Additionally, a significant (*p* < 0.01) decrease in body weight during 11 d of continuous oral administration of DEXA was observed compared to xylene and intact vehicle control mice (data not shown).

#### Ear examination findings

Noticeable ear redness and edoema associated with an acute inflammatory response (Cho et al. 2008; Lee and Ku 2008) was detected in xylene control mice two hours after topical application of xylene. However, these gross xylene-induced edoema and redness were dose-dependently inhibited after 11 d of continuous oral treatment with KOG 100, 200, and 400 mg/kg compared to xylene control mice. In addition, DEXA treated mice also demonstrated visual decreases in edoema and ear redness compared to xylene control mice. KOG 200 favourably inhibited xylene-induced edoema and ear redness relative to DEXA (1 mg/kg; data not shown).

#### Changes on the ear weights

**Figure 2.**
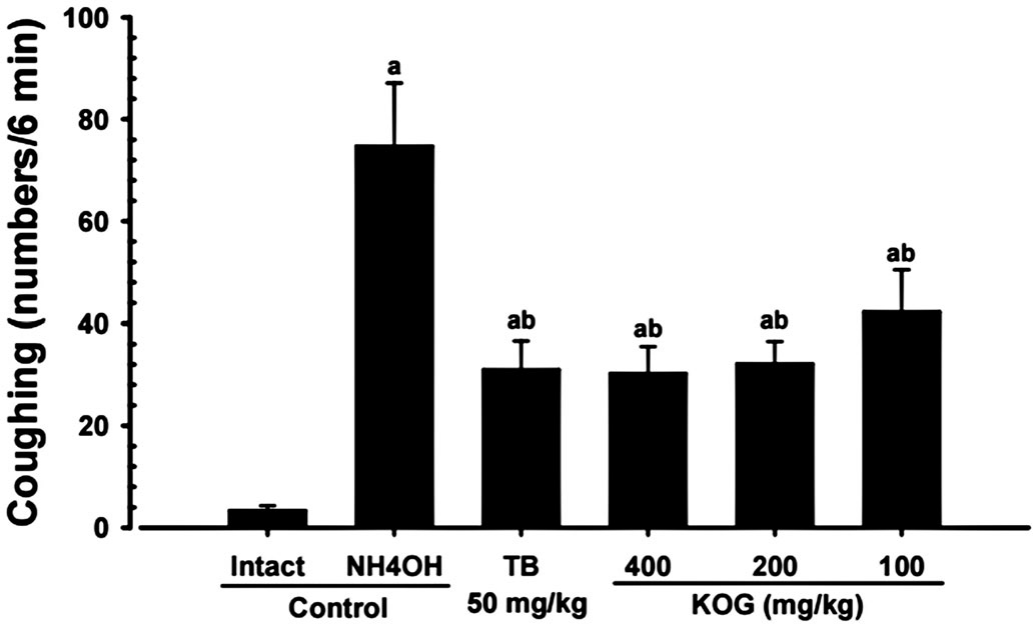
Ear weights during anti-inflammatory assay. Values are expressed as means ± SD of eight mice. KOG: *Kyeongok-go*, Traditional mixed herbal formulation. DEXA: Dexamethasone. *F*-value = 41.20 (absolute weights) and 34.56 (relative weights). ^a^*p* < 0.01 as compared with intact control by LSD test. ^b^*p* < 0.01 as compared with xylene control by LSD test.

Significant (*p* < 0.01) increases in the relative and absolute weights of each ear were observed in xylene control mice compared to intact vehicle control mice 2 h after topical application of xylene. Nevertheless, significant (*p* < 0.01) and dose-dependent decreases in the relative and absolute weights of each ear were observed with KOG 100, 200, and 400 mg/kg treatment relative to xylene control mice. In addition, DEXA treated mice also showed significant (*p* < 0.01) decreases in the relative and absolute weights of each ear compared to xylene control mice. KOG 200 favourably inhibited the effects of xylene on the relative (F value = 34.56) and absolute weights (F value = 41.20) of mouse ears compared to DEXA (1 mg/kg) treatment (Figure 2).

#### Histopathological analyses of ear

**Figure 3.**
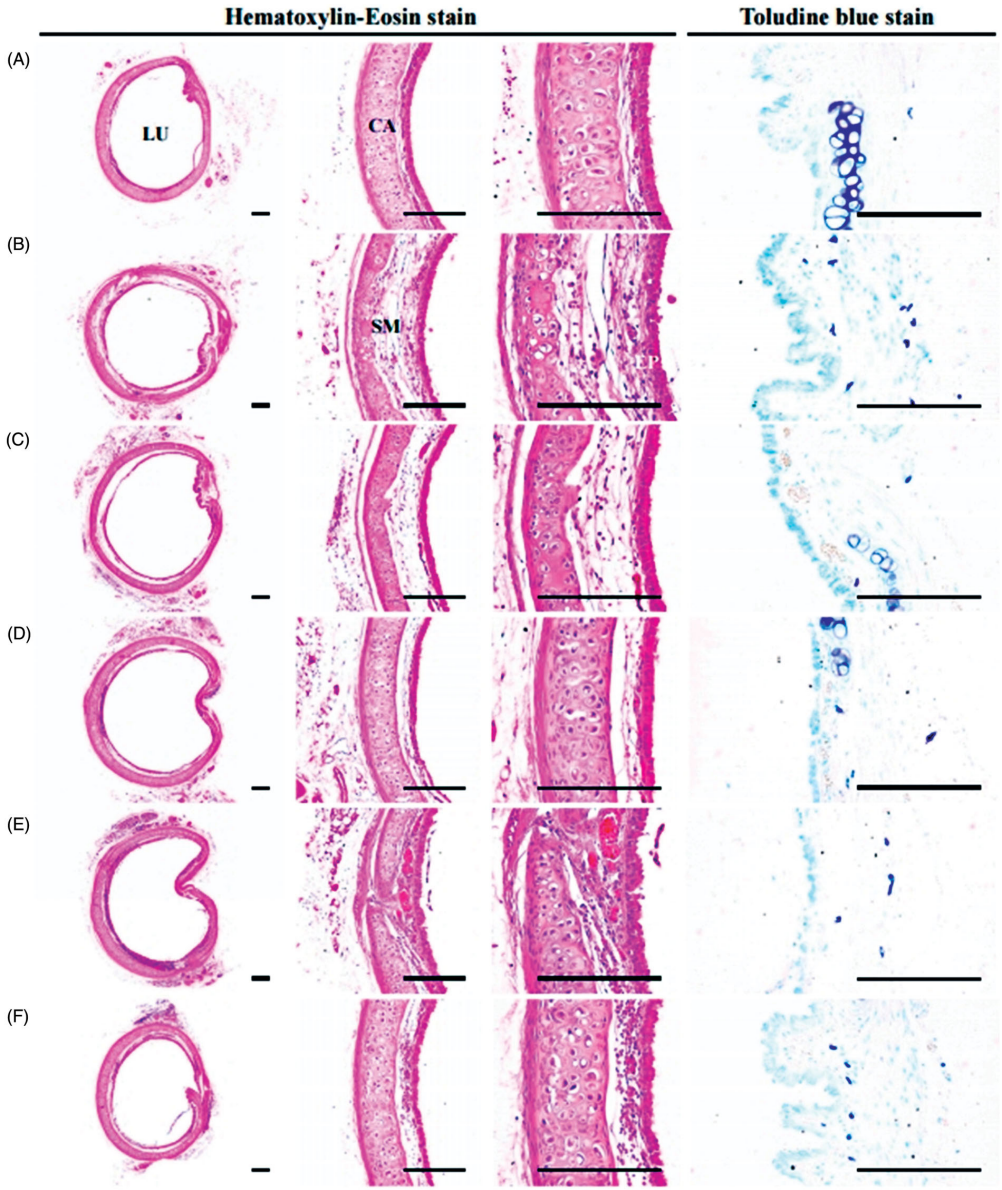
Representative histological images of ear during anti-inflammatory assay. (A) Intact vehicle control: Vehicle (distilled water) treated intact control mice. (B) Xylene control: Vehicle administered and xylene topically applied control mice. (C) DEXA: DEXA 1 mg/kg administered and xylene topically applied mice. (D) KOG100: KOG 100 mg/kg administered and xylene topically applied mice. (E) KOG200: KOG 200 mg/kg administered and xylene topically applied mice. (F) KOG400: KOG 400 mg/kg administered and xylene topically applied mice. KOG: *Kyeongok-go*, Traditional mixed herbal formulation. DEXA: Dexamethasone; AS: Anterior surface; EP: Epidermis; DE: Dermis; CA: Cartilage. Scale bars = 120 µm.

Significant (*p* < 0.01) increases in the total thickness and thickness of the dermis were observed in xylene control mice. Increases in the infiltrating inflammatory cell numbers in the dermis, decreases in dermis collagen fibre occupied regions without significant changes in the epidermis of the ear and degranulation related decreases in mast cells in the dermis were observed in xylene control mice; and were defined as the classical acute contact inflammation-dermatitis associated histopathological findings (Cho et al. 2008; Lee and Ku 2008). However, the dermatitis related histopathological findings were significantly (*p* < 0.01) and dose-dependently inhibited by 11 d of continuous oral pre-treatment with KOG 100, 200, and 400 mg/kg compared to xylene control mice. In addition, DEXA also significantly (*p* < 0.01) reduced the symptoms of xylene-induced acute contact dermatitis compared to xylene control mice. KOG 400 had a somewhat lower inhibitory effect on the induction of the histopathological findings observed in response to xylene-induced acute contact relative to DEXA (1 mg/kg; Table 2, Figure 3).

**Table 2.** Histomorphometry of ear in anti-inflammatory assay.

Groups	Controls		DEXA 1 mg/kg	AR			F or H values
Index	Intact	Xylene		400 mg/kg	200 mg/kg	100 mg/kg	
Total thickness (μm)	104.71 ± 12.63	266.25 ± 27.90^d^	100.15 ± 10.50^e^	158.47 ± 15.11^d^^e^	165.56 ± 12.94^d^^e^	184.18 ± 22.08^d^^e^	40.90
Epidermis thickness (μm)	9.02 ± 1.04	8.96 ± 1.26	8.45 ± 1.77	8.70 ± 0.92	8.59 ± 0.70	8.85 ± 0.78	0.29
Dermis thickness (μm)	55.83 ± 11.72	133.52 ± 22.90^a^	53.45 ± 15.45^c^	70.34 ± 4.91^bc^	84.99 ± 12.52^a^^c^	90.94 ± 12.26^a^^c^	34.12
IF cells (numbers/mm^2^)	14.25 ± 4.65	266.88 ± 55.98^d^	20.13 ± 10.91^e^	68.25 ± 13.39^d^^e^	90.50 ± 24.11^d^^e^	141.88 ± 23.41^d^^e^	43.31
Mast cells (numbers/mm^2^)	69.38 ± 17.43	8.75 ± 4.40^d^	61.63 ± 14.31^e^	41.38 ± 6.80^d^^e^	27.63 ± 6.93^d^^e^	24.13 ± 4.85^d^^e^	41.51
Collagen fibres (%/mm^2^)	78.83 ± 10.74	25.82 ± 7.10^a^	76.95 ± 13.65^c^	68.53 ± 4.80^bc^	64.68 ± 9.21^a^^c^	52.64 ± 10.03^a^^c^	33.39

Values are expressed mean ± SD of eight mice.

DEXA: Dexamethasone; KOG: *Kyeongok-go*, Traditional mixed herbal formulation; IF: inflammatory.

^a^*p* < 0.01 and ^b^*p* < 0.05 as compared with intact control by LSD test.

^c^*p* < 0.01 as compared with xylene control by LSD test.

^d^*p* < 0.01 as compared with intact control by MW test.

^e^*p* < 0.01 as compared with xylene control by MW test.

### Expectorant assay

#### Body weight changes

No significant changes in the body weights of KOG 100, 200, and 400 mg/kg treated mice, or AM (250 mg/kg) treated mice compared to intact vehicle control mice, were observed (data not shown).

#### Body surface gross examination findings

Visible dose-dependent increases in body redness were observed with KOG 100, 200, and 400 mg/kg treatment compared to intact vehicle control mice, suggesting an increase in phenol red secretion and uptake. In addition, a dramatic increase in body redness 30 min after intraperitoneal injection of phenol red compared to intact control mice was also observed in AM treated mice. KOG 200 treatment resulted in a significant increase in body surface redness compared to AM (250 mg/kg) treatment (data not shown).

#### Changes in mucous secretion

**Figure 4.**
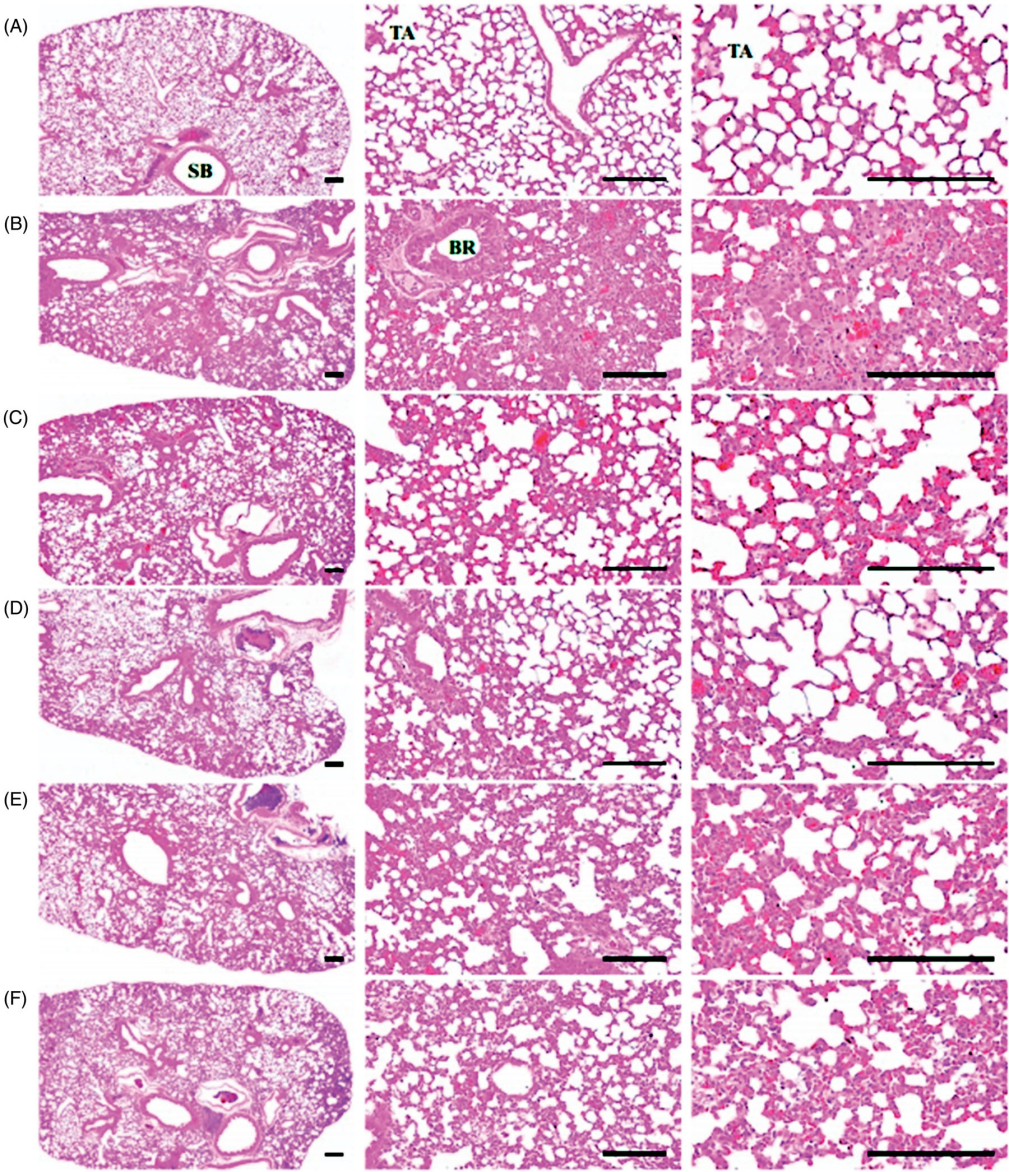
Mucous secretions during the expectorant assay. Values are expressed as means ± SD of eight mice. KOG: *Kyeongok-go*, Traditional mixed herbal formulation. AM: Ambroxol; OD: Optical density; TLF: Trachea lavage fluid. *F*-value = 14.45. ^a^*p* < 0.01 as compared with intact control by LSD test.

Significant (*p* < 0.01) and dose-dependent increases in the TLF OD values were observed 30 min after intraperitoneal injection of phenol red in KOG 100, 200, and 400 mg/kg treatments compared to intact vehicle control mice, suggesting an increase in tracheal mucous secretion (Engler and Szelenyi 1984; Zhang et al. 2009; Wang et al. 2012). In addition, AM treated mice also showed significant (*p* < 0.01) increases in TLF OD values relative to intact vehicle control mice. KOG 200 treatment resulted in the most favourable increase (F value = 14.45) in mucous secretion compared to AM (250 mg/kg) treatment (Figure 4).

#### Histopathological findings in the intrapulmonary secondary bronchi

**Figure 5.**
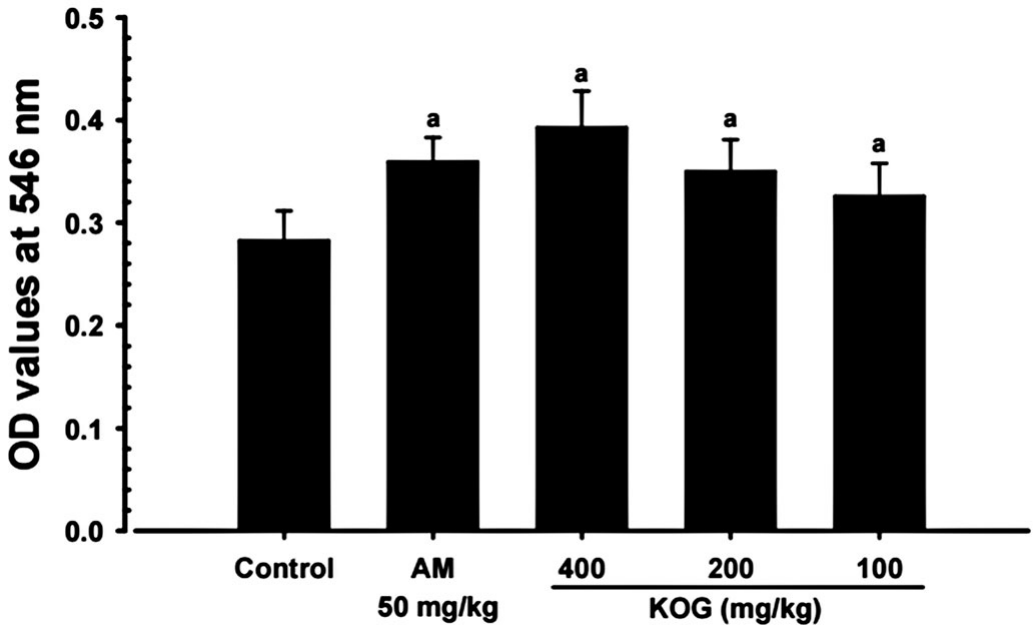
Representative histological images of intrapulmonary secondary bronchus during expectorant assay. (A) Intact vehicle control: Vehicle (distilled water) treated intact control mice. (B) AM: AM 250 mg/kg administered mice. (C) AR100: KOG 100 mg/kg administered mice. (D) AR200: KOG 200 mg/kg administered mice. (E) AR400: KOG 400 mg/kg administered mice. KOG: *Kyeongok-go*, Traditional mixed herbal formulation. AM: Ambroxol; PAS: Periodic acid Schiff stain; LU: Lumen; EP: Epithelium. Scale bars = 60 µm.

**Figure 6.**
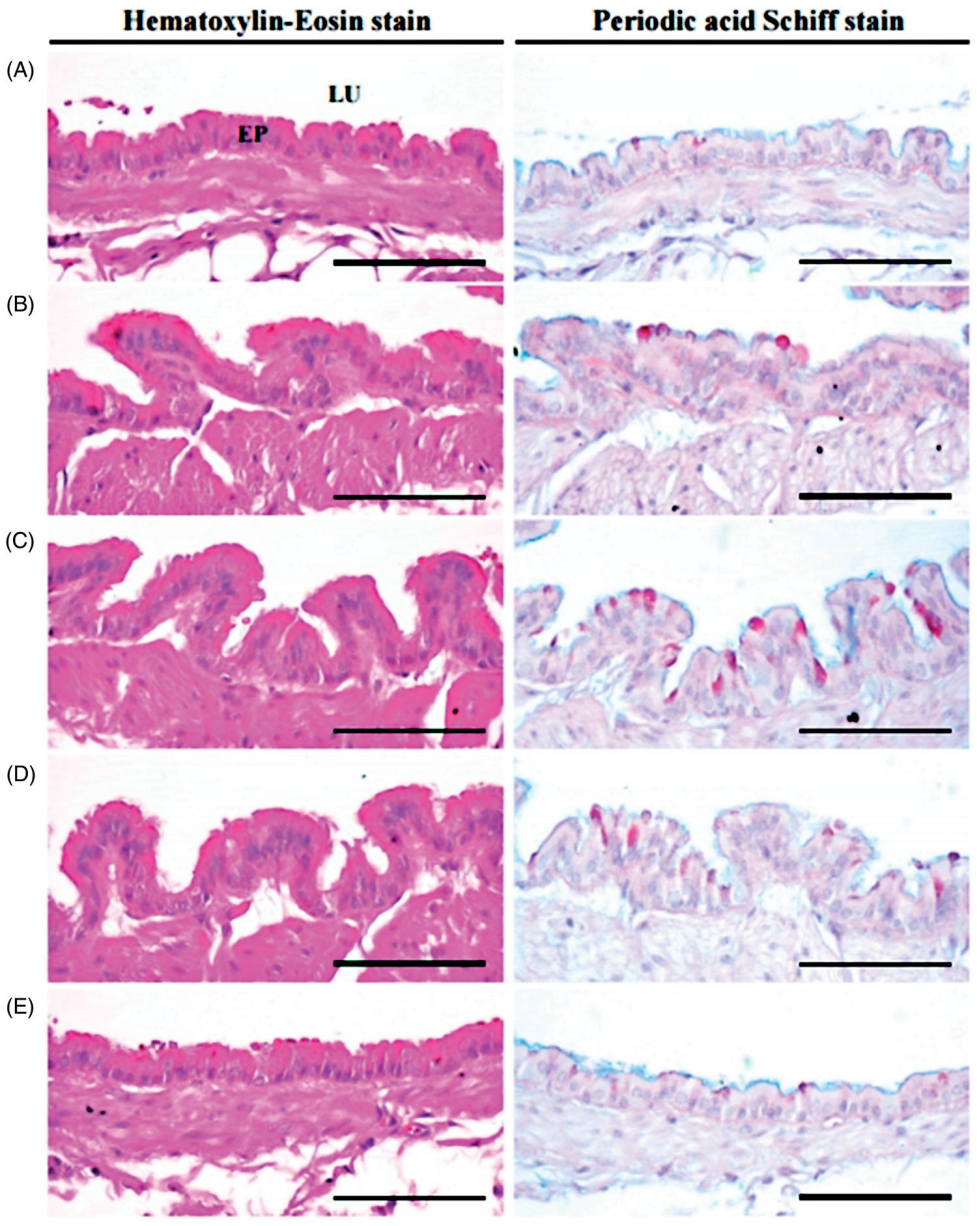
Changes on the intrapulmonary secondary bronchus epithelial thicknesses and PAS-positive mucous producing cell numbers during expectorant assay. Values are expressed as means ± SD of eight mice. KOG: *Kyeongok-go*, Traditional mixed herbal formulation. AM: Ambroxol; PAS: Periodic acid Schiff stain. *H*-value = 25.41 (bronchus epithelial thicknesses) and 23.80 (mucous producing cell numbers). ^a^*p* < 0.01 and ^b^*p* < 0.05 as compared with intact control by MW test.

A significant (*p* < 0.05) and dose-dependent increase in the number of PAS positive mucous producing cells and in the thickness of the intrapulmonary secondary bronchi mucosa were observed with KOG 100, 200, and 400 mg/kg treatments relative to intact vehicle control mice. This suggests that mucous secretion (or activity of bronchus mucosa) significantly increased in response to KOG treatment (Engler and Szelenyi 1984; Zhang et al. 2009; Wang et al. 2012). In addition, the thickness of the intrapulmonary secondary bronchi mucosa and the number of PAS positive mucous producing cell were also significantly (*p* < 0.01) increased in AM treatment compared to intact vehicle control mice. KOG 200 and AM (250 mg/kg) treatment resulted in similar PAS positive mucous producing cells and intrapulmonary secondary bronchi mucosa thickness (H values are 25.41 and 23.80, respectively; Figures 5 and 6).

### Antitussive assay

#### Body weight changes

No significant differences between the body weights of intact vehicle control and NH_4_OH control mice were observed during the treatment period; or in KOG 100, 200, 400 and TB treated mice, relative to NH_4_OH control mice (data not shown).

#### Changes in cough frequency

**Figure 7.**
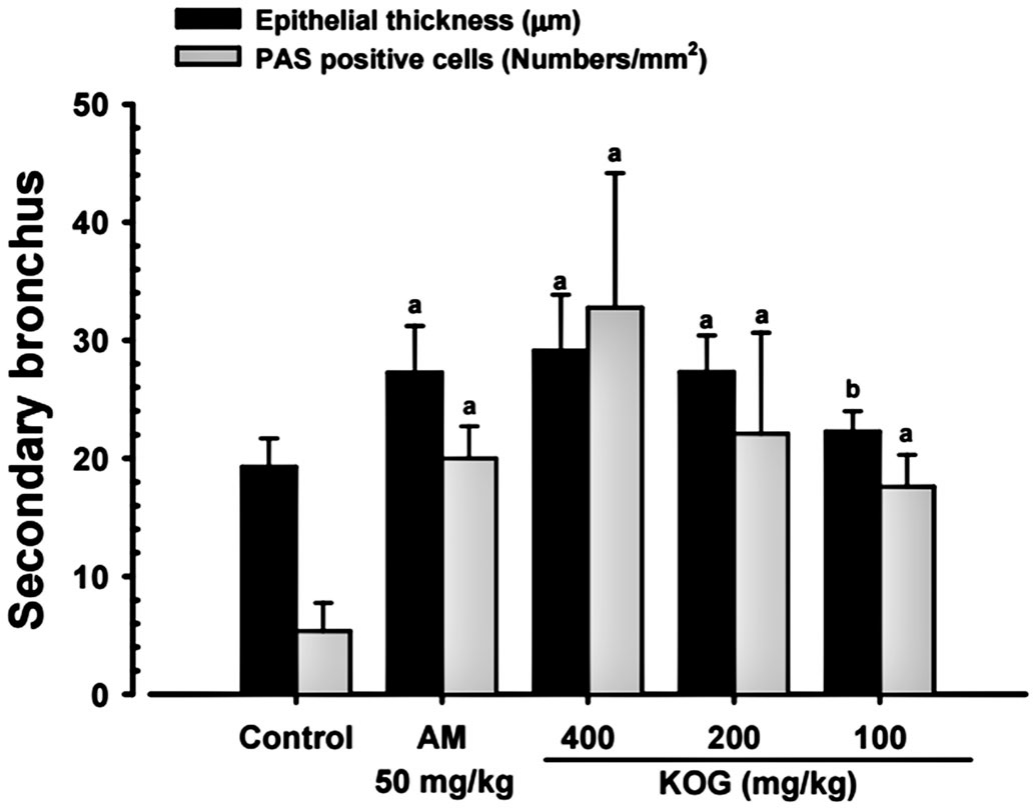
Coughing frequency during the antitussive assay. Values are expressed as means ± SD of eight mice. NH_4_OH: Ammonia hydroxide; KOG: *Kyeongok-go*, Traditional mixed herbal formulation; TB: Theobromine. *H*-value = 38.80. ^a^*p* < 0.01 as compared with intact control by MW test. ^b^*p* < 0.01 as compared with NH_4_OH control by MW test.

A significant (*p* < 0.01) increase in the number of coughs within a six min interval after a 45 sec exposure to NH_4_OH was observed in NH_4_OH control mice than in the intact vehicle control mice. Whereas, a significant (*p* < 0.01) and dose-dependent decrease in coughing was observed in KOG 100, 200, and 400 mg/kg compared to NH_4_OH control mice. In addition, TB treated mice also showed significant (*p* < 0.01) decreases in the number of coughs compared to NH_4_OH control mice. KOG 200 favourably inhibited (H value = 38.80) NH_4_OH-induced coughing compared to treatment with TB (50 mg/kg; Figure 7).

#### Histopathological findings on the trachea and lung

**Figure 8.**
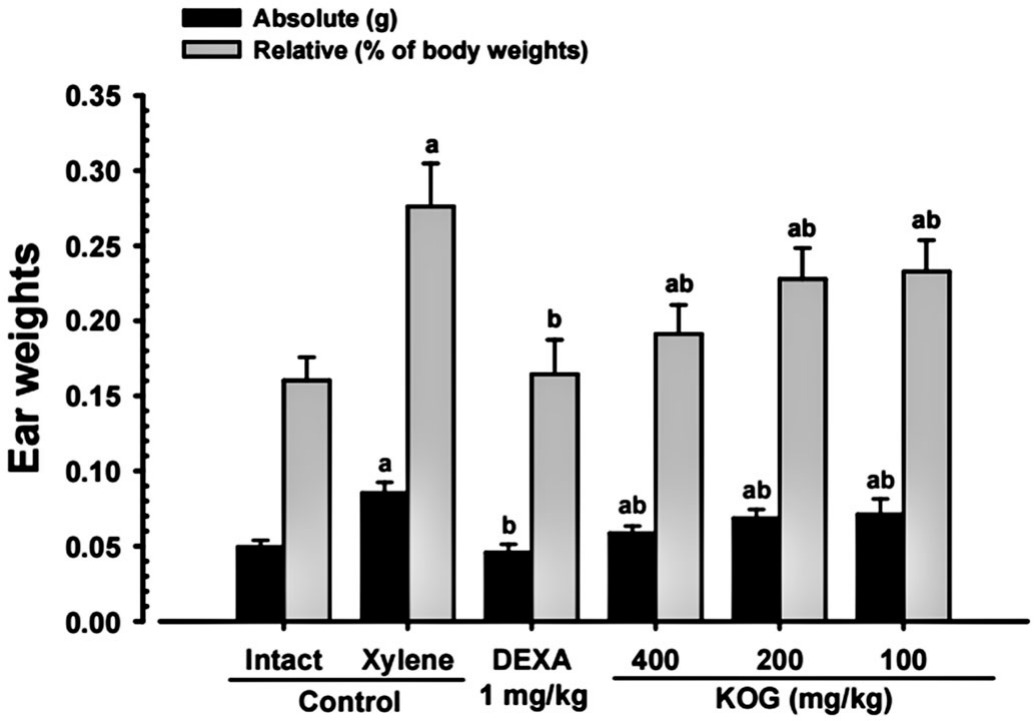
Representative histological images of trachea during antitussive assay. (A) Intact vehicle control: Vehicle (distilled water) treated intact control mice. (B) NH_4_OH control: Vehicle administered and NH_4_OH exposed control mice. (C) TB: TB 50 mg/kg administered and NH_4_OH exposed mice. (D) KOG100: KOG 100 mg/kg administered and NH_4_OH exposed mice. (E) KOG200: KOG 200 mg/kg administered and NH_4_OH exposed mice. (F) KOG400: KOG 400 mg/kg administered and NH_4_OH exposed mice. NH_4_OH: Ammonia hydroxide; KOG: *Kyeongok-go*, Traditional mixed herbal formulation; TB: Theobromine; LU: Lumen; EP: Epithelium; SM: Submucosa; CA: Cartilages. Scale bars = 120 µm.

**Figure 9.**
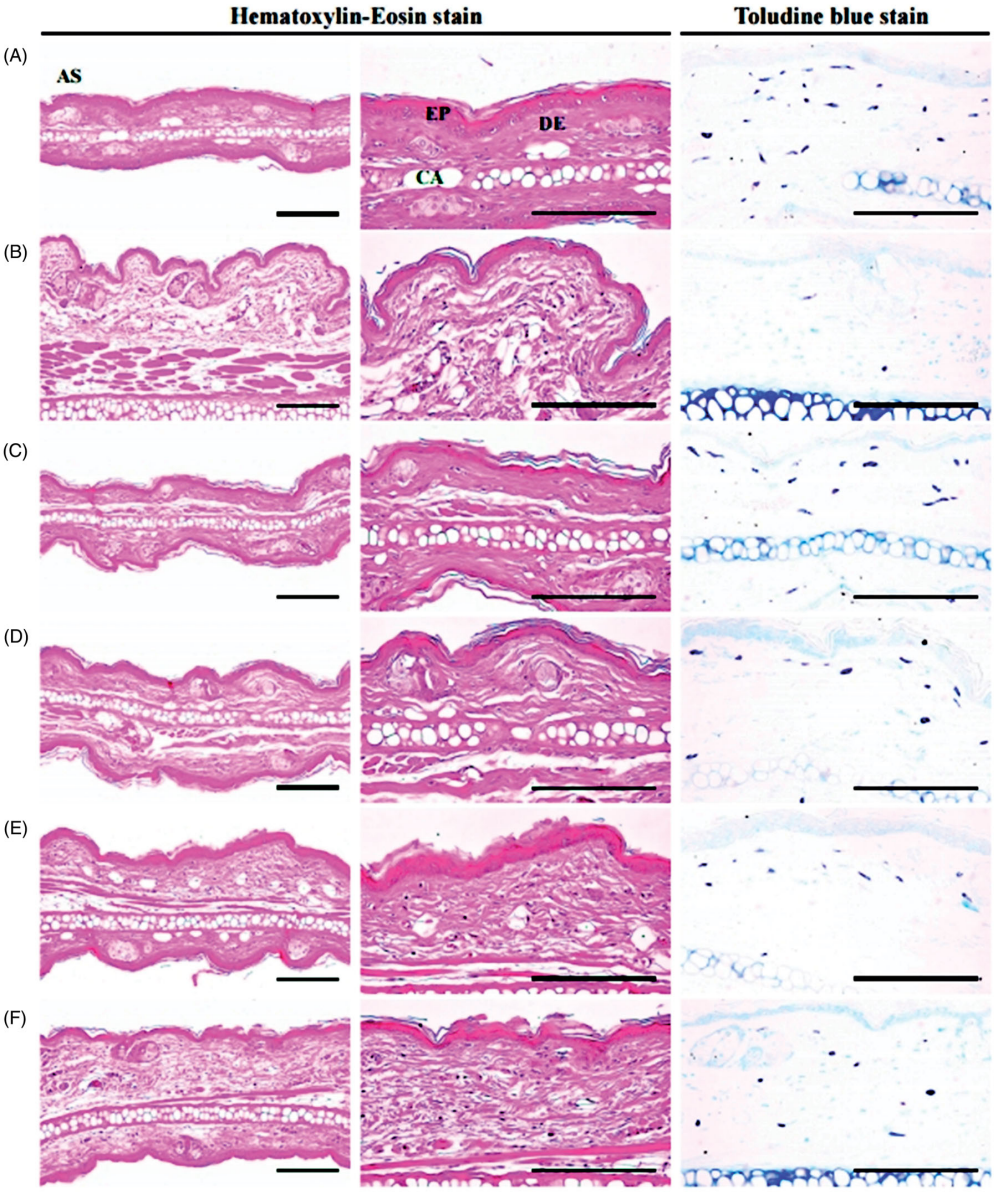
Representative histological images of lung in antitussive assay. (A) Intact vehicle control: Vehicle (distilled water) treated intact control mice. (B) NH_4_OH control: Vehicle administered and NH_4_OH exposed control mice. (C) TB: TB 50 mg/kg administered and NH_4_OH exposed mice. (D) KOG100: KOG 100 mg/kg administered and NH_4_OH exposed mice. (E) KOG200: KOG 200 mg/kg administered and NH_4_OH exposed mice. (F) KOG400: KOG 400 mg/kg administered and NH_4_OH exposed mice. NH_4_OH: Ammonia hydroxide. KOG: *Kyeongok-go*, Traditional mixed herbal formulation; TB: Theobromine; ASA: Alveolar surface area; SB: Secondary bronchus; BR: Bronchus; TA: Alveolus-terminal bronchiole. All Haematoxylin-Eosin stain. Scale bars: 120 µm.

A significant (*p* < 0.01) increase in the total tracheal wall, epithelium, and submucosa thickness and decrease in the diameter of the tracheal lumen was observed. The number of infiltrating mast and inflammatory cells increased, ASA decreased, and increases in the number of inflammatory cells in the alveolar septum and the thickness of the alveolar septum were observed in the lungs and trachea of NH_4_OH control mice. These are classical histopathological findings associated with acute allergic inflammation (Lebargy et al. 1987; Choi et al. 2005; Ku et al. 2012). Conversely, these NH_4_OH-induced acute allergic inflammation histopathological findings were significantly (*p* < 0.01) and dose-dependently inhibited by day 11 of continuous oral pre-treatment with KOG 100, 200 and 400 mg/kg, relative to NH_4_OH control mice. Furthermore, TB significantly (*p* < 0.01) reduced the pathologies associated with NH_4_OH-induced acute allergic inflammation relative to NH_4_OH control mice. Treatment with KOG 200 mg/kg significantly reduced the histopathological findings associated with NH_4_OH-induced acute allergic inflammation relative to TB (50 mg/kg; Table 3, Figures 8 and 9).

**Table 3.** Histomorphometry of trachea and lung in antitussive assay.

Groups	Controls		TB 50 mg/kg	AR			F or H values
Index	Intact	NH_4_OH		400 mg/kg	200 mg/kg	100 mg/kg	
Trachea							
Diameter of lumen (μm)	1149.31 ± 130.31	631.47 ± 100.10^a^	867.88 ± 112.58^a^^c^	935.22 ± 91.87^a^^c^	907.45 ± 71.84^a^^c^	834.89 ± 102.06^a^^c^	21.09
Total wall thickness (μm)	163.74 ± 16.46	220.52 ± 13.35^a^	190.12 ± 10.84^a^^c^	184.00 ± 10.90^a^^c^	188.63 ± 8.85^a^^c^	193.78 ± 11.86^a^^c^	17.76
Epithelium thickness (μm)	14.43 ± 2.33	39.23 ± 10.38^d^	25.10 ± 4.44^d^^e^	23.14 ± 1.88^d^^e^	25.30 ± 2.91^d^^e^	27.62 ± 2.84^d^^e^	35.89
Submucosa thickness (μm)	26.99 ± 4.55	91.97 ± 10.56^a^	48.16 ± 12.56^a^^c^	39.51 ± 7.17^bc^	49.78 ± 12.92^a^^c^	63.10 ± 13.50^a^^c^	34.93
IF cells (numbers/mm^2^)	19.50 ± 9.90	460.38 ± 105.94^d^	182.50 ± 26.77^d^^e^	189.50 ± 18.53^d^^e^	198.63 ± 16.13^d^^e^	286.75 ± 65.91^d^^e^	39.48
Mast cells (numbers/mm^2^)	1.38 ± 0.74	37.25 ± 12.62^d^	14.88 ± 4.26^d^^e^	8.50 ± 1.60^d^^e^	13.75 ± 4.46^d^^e^	16.38 ± 5.71^d^^e^	38.97
Lung – alveolar regions							
ASA (%/mm^2^)	76.65 ± 9.12	30.18 ± 10.58^a^	51.77 ± 6.57^a^^c^	51.06 ± 8.52^a^^c^	51.96 ± 7.56^a^^c^	43.85 ± 4.11^a^^c^	28.53
Septum thickness (μm)	6.87 ± 1.14	71.20 ± 11.68^d^	30.85 ± 11.34^d^^e^	27.82 ± 3.88^d^^e^	30.96 ± 8.18^d^^e^	43.65 ± 10.69^d^^e^	36.32
IF cells (numbers/mm^2^)	58.13 ± 21.62	1808.63 ± 394.30^d^	508.75 ± 114.22^d^^e^	472.75 ± 105.10^d^^e^	513.13 ± 86.44^d^^e^	1221.13 ± 235.65^d^^e^	40.41

Values are expressed mean ± SD of eight mice.

NH_4_OH: Ammonia hydroxide; TB: Theobromine; KOG: *Kyeongok-go*, Traditional mixed herbal formulation; IF: inflammatory; ASA: Alveolar surface area.

^a^*p* < 0.01 and ^b^*p* < 0.05 as compared with intact control by LSD test.

^c^*p* < 0.01 as compared with NH_4_OH control by LSD test.

^d^*p* < 0.01 as compared with intact control by MW test.

^e^*p* < 0.01 as compared with NH_4_OH control by MW test.

## Discussion

In this study, the anti-inflammatory, expectorant, and antitussive properties of KOG were analyzed using the appropriate animal models in order to facilitate the development of natural therapies for various respiratory disorders.

All mice analyzed, except for DEXA treated mice in the xylene-induced acute inflammatory mouse ear model, showed no differences in body weights during the 11 d treatment period, compared to age-matched normal control mice (Fox et al. 1984; Tajima 1989). Therefore, 11 d continuous oral pre-treatment of KOG (100, 200, and 400 mg/kg) did not critically affect the body weights of mice. DEXA treated mice in the xylene-induced acute inflammatory mouse ear model showed significant decreases in body weight 2 d after the initial administration of DEXA and body weight increases during the 11 d of continuous oral administration of DEXA were observed as relative to xylene and intact vehicle control mice. The application of DEXA has been associated with the decrease in body weight in various anti-inflammatory animal studies (Kim et al. 2010; Ku et al. 2012).

Linctus (a cough medication in syrup form) is used to treat cough and its related symptoms. For dry cough, treatments with antitussives or cough suppressants are used to suppress the body's urge to cough. Whereas, in productive coughs (producing phlegm), expectorants are used to loosen mucus from the respiratory tract (Smith et al. 2008; Dicpinigaitis 2012). Animal models including mice can be used to verify the antitussive effects of drugs by detecting and counting the number of coughs produced by tussive stimulus, like NH_4_OH given to animals, and by comparing the number of coughs induced in response to the administration of tussive, as an effective and simple experimental approach (Zhang et al. 2009; Wang et al. 2012). In this study, the antitussive effects of KOG were tested using the NH_4_OH exposure coughing mouse model. ASA is directly related to the gas exchange capacity of lung, the higher the ASA, the higher the gas exchange capacity (Davey et al. 2002; Choi et al. 2005; Ku et al. 2012).

The results of this experiment, marked significant increases in the frequency of coughs, decreases of the diameter of tracheal lumen, increases in the total tracheal wall, epithelium and submucosa thickness, increases in the number of tracheal infiltrating mast and inflammatory cells, decreases in ASA, increases in the alveolar septum thickness, and inflammatory cell number in the alveolar septum were observed in NH_4_OH control as classic acute allergic inflammation with coughing (Lebargy et al. 1987; Choi et al. 2005; Ku et al. 2012). However, these NH_4_OH-induced acute allergic inflammation with coughing responses were significantly and dose-dependently inhibited by 11 d of continuous oral pre-treatment with KOG (100, 200, and 400 mg/kg), and KOG (200 mg/kg) showed favourable antitussive effects relative to TB (50 mg/kg) during NH_4_OH exposure. This is direct evidence that KOG (100, 200, and 400 mg/kg) favourably and potently exerts antitussive effects, at least within the parameters of this study.

Expectorants commonly increase the hydration or number of secretions, resulting in clearer secretions and as a byproduct lubricate the irritated respiratory tract, and effectively relieve various respiratory disorders (Gonzalez Garay et al. 2014; Rubin 2015). The expectorant properties of drugs have been evaluated by investigating the ability to produce mucus, one of the mechanisms of expectorants, and the tracheal phenol red secretion method has been utilized as a simple and an effective experimental approach (Engler and Szelenyi 1984; Zhang et al. 2009; Wang et al. 2012). The expectorant effects of KOG were detected using the phenol red secretion mouse method. The higher intensity of PAS (a special and classic histochemistrical stain to detect mucous and mucous producing cells) correlates with an increased activity of mucous producing cells (Tam et al. 2014; Honda et al. 2016).

After 11 d of continuous oral pre-treatment of KOG (100, 200, and 400 mg/kg), dose-dependent increased the OD values in TLF, intrapulmonary secondary bronchus mucosa thickness, body surface redness, and PAS positive mucous producing cells compared to vehicle control mice. This suggests that KOG has enhanced the expectorant properties by increasing the secretion of mucous or activity of the bronchus and trachea mucosa (Engler and Szelenyi 1984; Zhang et al. 2009; Wang et al. 2012). KOG (200 mg/kg) showed favourable expectorant effects compared to AM (250 mg/kg) in the tracheal phenol red secretion mouse model. These findings are direct evidence that KOG (100, 200, and 400 mg/kg) have favourable and obvious expectorant properties.

The xylene-induced acute inflammatory mouse ear model has commonly been employed as one of the traditional methods for identifying the efficacy of anti-inflammatory agents (Cho et al. 2008; Lee and Ku 2008). This animal model provides easy and simple verification method for anti-inflammatory effects of drugs by comparing the ear edoema, and changes in ear thickness, weight, and histopathology (Habashy et al. 2005; Cho et al. 2008; Lee and Ku 2008). The changes to the collagen fibre occupied region percentages in the dermis at histopathological inspections are directly linked to sclerosis of the skin or inflammatory oedematous changes, skin sclerosis (the increase in the percentages of collagen fibre occupied regions in the dermis), controversially the dermis inflammatory oedematous (the decrease in the percentages of collagen fibre occupied regions in the dermis) changes (Kim, Kang et al. 2015, Kim, Park et al. 2015).

In the current study, xylene-induced acute inflammatory mouse ear model was also used to identify the dose-dependent anti-inflammatory effects of KOG. Xylene control mice showed noticeable signs of redness or ear edoema, significant increases in ear dermis and total thickness ear weight, degranulation related decrease of mast cells in the dermis, the number of infiltrating inflammatory cells on the ear dermis, decrease in dermis collagen fibre occupied regions, without significant changes on the thickness of the epidermis of the war, suggestive of acute contact inflammation-dermatitis (Cho et al. 2008; Lee and Ku 2008). However, these signs of xylene-induced contact dermatitis were significantly and dose-dependently inhibited by 11 d of continuous oral pre-treatment with KOG 100, 200, and 400 mg/kg. KOG (400 mg/kg) treatment resulted in slight but favourable anti-inflammatory activity compared to DEXA treatment in the xylene-induced acute inflammatory mouse ear model. These findings are considered direct evidence that KOG (100, 200, and 400 mg/kg) have positive and clear anti-inflammatory impacts.

Mast cells, widely distributed throughout the body, play a crucial role in a variety of inflammatory and allergic disorders. They have cell surface receptors for immunoglobulin E (IgE) which are activated by the interaction of antigen specific IgE bound to the receptors and its antigen (Holgate 2000). After activation, these cells release various bioactive substances including lipid and histamine mediators, which cause an immediate type I allergic reaction. Mast cell degranulation increases at the acute (Gauvreau et al. 2000) and chronic stages (Chanez et al. 1993; Armbrust et al. 1997) of a variety of allergic and inflammatory diseases. During acute inflammation, marked depletion of mast cells due to increases in mast cell activation, and degranulation in the dermis have been detected, and therefore, the inhibition of mast cell activation has been used as a valuable index to predict the efficacy of anti-inflammatory drugs (Cha et al. 2013; Yang et al. 2013).

Apart from the dermis, it has also been reported that increases in mast cells due to migration from nearby connective tissues have been reported in cases of various allergic respiratory disorders (Dahlin et al. 2011; Stankevicius et al. 2012). Noticeable migration related increase in the mean number of mast cells in the NH_4_OH control mouse of the NH_4_OH exposure coughing mouse model and significant decreases in the mean infiltrating mast cell counts in the ear dermis were observed, which might have resulted from degranulation as demonstrated in the xylene control of the xylene-induced acute inflammatory mouse ear model. However, these abnormal changes in mast cells were dose-dependently and favourably normalized by 11 d of uninterrupted oral pre-treatment of KOG (100, 200, and 400 mg/kg). This is direct evidence that the antitussive effects of KOG on NH_4_OH exposure, and the anti-inflammatory effects observed in response to xylene may have occurred by controlling mast cell activation and regulating degranulation or migration.

## Conclusions

The results of the present study suggested that KOG should be considered as a therapeutic agent for the treatment of a variety of respiratory diseases, particularly those caused by exposure to environmental toxins.

## Data Availability

The supporting data could be provided from the corresponding author upon request.
